# Surficial carbonates in mantle plume sources revealed by magnesium isotopes

**DOI:** 10.1126/sciadv.aee9247

**Published:** 2026-10-09

**Authors:** Ze-Zhou Wang, Fang-Zhen Teng, Matthew G. Jackson, Catherine Chauvel, Cédric Hamelin

**Affiliations:** ^1^ State Key Laboratory of Geological Processes and Mineral Resources, China University of Geosciences, Beijing 100083, China.; ^2^ Isotope Laboratory, Department of Earth and Space Sciences, University of Washington, Seattle, WA 98195, USA.; ^3^ Department of Earth Science, University of California, Santa Barbara, Santa Barbara, CA 93106, USA.; ^4^ Université Paris Cité, Institut de Physique du Globe de Paris, CNRS, 75005 Paris, France.; ^5^ Independent Scholar, Søndre Skogveien 7, 5055 Bergen, Norway.

## Abstract

Deep recycling of surficial carbonates into the mantle may play a critical role in shaping Earth’s long-term habitability, yet its contribution to the deep mantle carbon budget remains uncertain. Magnesium isotopes provide a promising tracer for addressing this issue, because of the uniquely light isotopic signatures of sedimentary carbonates. Here, we present high-precision magnesium isotopic compositions (δ^26^Mg) for 152 globally distributed plume-related lavas sampling diverse mantle end-members. We find that δ^26^Mg values below the bulk silicate Earth range are commonly associated with increasingly pronounced radiogenic isotopic signatures indicative of recycled crustal components. These features are best explained by the incorporation of isotopically light magnesium derived from subducted carbonate-bearing crustal materials into their mantle sources. On the basis of these observations, the lower mantle is estimated to contain up to ∼130 or ∼230–parts per million recycled carbon, potentially constituting a substantial portion of its total carbon budget.

## INTRODUCTION

The deep carbon cycle driven by slab subduction and volcanic degassing potentially governs Earth’s long-term climate stability and habitability over millions to billions of years (*1*). Approximately 85% of subducting carbon is estimated to be hosted in carbonates (i.e., calcite, aragonite, and dolomite) within sediments and altered oceanic crust (AOC) (*2*). Therefore, the fate of subducted carbonates is critical for understanding the efficiency of deep carbon recycling. However, existing models diverge widely, with some suggesting nearly complete liberation of subducted carbonates into the shallow upper mantle [e.g., (*3*, *4*)], whereas others argue for only partial loss during subduction-zone devolatilization, thereby allowing deep subduction of carbonates [e.g., (*5*)]. Resolving this debate remains challenging because direct evidence for recycled carbonates in the deep mantle is mostly limited to superdeep diamonds and their inclusions (*6*).

Traditionally, studies of the deep carbon cycle have relied heavily on isotopes of carbon and noble gases (*7*, *8*), which have provided critical evidence for the presence of recycled carbon in the mantle. Carbon isotopes, in particular, are effective in distinguishing between organic carbon (δ^13^C as low as ∼−30‰, where δ^13^C is the deviation of the ^13^C/^12^C ratio relative to that of Pee Dee belemnite) and sedimentary inorganic carbon (δ^13^C = ∼−2 to 0‰) (*9*). However, inorganic carbonates are not significantly isotopically different from the mantle (δ^13^C = ∼−5‰) (*9*). Furthermore, carbon isotopes are prone to fractionation by degassing during magma ascent and volcanic eruption. These limitations hinder the effectiveness of using magma δ^13^C to identify recycled inorganic carbonates in mantle sources. By contrast, the isotopes of carbonate-forming cationic elements (e.g., Mg, Ca, and Zn) are able to overcome these shortcomings owing to the nonvolatile nature of these elements and marked isotopic contrast between marine carbonates and the mantle (*10*–*13*).

Among these novel isotope tracers, Mg isotopes have emerged as a particularly powerful tool, as marine carbonates have uniquely low δ^26^Mg values (the deviation of the ^26^Mg/^24^Mg ratio relative to that of DSM3 Mg standard; −5 to −1‰), compared with the bulk mantle or bulk silicate Earth (BSE; δ^26^Mg = −0.25 ± 0.04‰) and other subducted lithologies, including serpentinites, carbonate-poor oceanic crust, and silicate-rich sediments, which typically have BSE-like or higher δ^26^Mg values (*14*). Thus, recycled carbonates can impart diagnostic light Mg isotopic signatures to the mantle or its derivative magmas. However, light Mg isotopic signatures have been rarely observed in arc lavas, which have BSE-like to higher δ^26^Mg values (*15*–*17*), although their mantle sources have been considered as a major reservoir of carbonate input from subducted slabs (*2*). This can be explained in two ways: (i) isotopically heavy Mg-bearing components, such as serpentinite-derived fluids or talc, may dominate Mg fluxes at subarc mantle, thereby masking isotopically light Mg contributed by recycled carbonates (*15*, *18*, *19*); (ii) carbonates released into subarc mantle during slab dehydration are dominated by Ca-rich species (*20*, *21*), which contain too little Mg to significantly modify the Mg isotopic composition of the mantle.

Magnesium-rich carbonates (e.g., dolomite and magnesite) are much less soluble in slab-derived fluids than calcites or aragonites (*20*–*22*) and can therefore survive slab dehydration and transport isotopically light Mg into the deep mantle. In addition, carbonates may undergo isotopic exchange with surrounding silicates in the slab during metamorphic reactions, transferring their isotopically light Mg to the latter (*23*). Consequently, the characteristic Mg isotopic signals of carbonates can be retained in deeply subducted sediments or AOC, as evidenced by the observations of low δ^26^Mg values in high/ultrahigh-pressure marbles and eclogites (*23*, *24*). The recent discovery of extremely low δ^26^Mg values (down to −1.4‰) in superdeep diamond-hosted ferropericlase inclusions provides direct evidence that isotopically light Mg, potentially derived from recycled carbonates, can be preserved in the lower mantle (*25*). These observations highlight the potential of Mg isotopes in tracing deep carbonate cycling beyond arc subduction zones.

Oceanic basalts provide one of the best examples to explore this potential. The chemical diversity of mid-ocean ridge basalts (MORBs) and oceanic island basalts (OIBs) indicates compositional heterogeneity within the convecting mantle. MORBs primarily sample the upper domain depleted in incompatible elements (those that preferentially enter melts during partial melting of the mantle), known as the depleted MORB mantle (DMM), whereas OIB sources entrained by mantle plumes derive from chemically and isotopically more heterogeneous reservoirs at greater depths, potentially near the core-mantle boundary (*26*–*29*). Variations in radiogenic isotopes categorize the OIB sources into several distinct mantle end-members, including HIMU (high U/Pb ratio), EM1 (enriched mantle type 1), and EM2 (enriched mantle type 2), which have been commonly interpreted to reflect recycling of surficial or near-surficial materials such as sediments, AOC, and subcontinental lithosphere into the deep mantle (*26*–*29*). Therefore, if subducted carbonates are transported into the lower mantle, then their characteristic light Mg isotopic signatures may be preserved in plume-fed OIBs. Nevertheless, low δ^26^Mg values are scarcely reported in OIBs. The only well-documented case is the EM1-type OIBs from Pitcairn Islands, which record the lowest value (−0.40‰) previously reported, interpreted as contributions from carbonate-bearing sediments (*30*). Beyond this region, most of the OIBs have δ^26^Mg values within the BSE range (*31*–*35*). Furthermore, the lack of paired radiogenic isotope data for most published Mg isotopic analyses of MORBs and OIBs hampers attributions of Mg isotopic variations to specific mantle end-members or recycled source components.

In this study, we conducted a comprehensive Mg isotopic investigation of 152 basalt samples from globally distributed hot spots and mid-ocean ridges (mostly from plume-proximal segments) (Fig. 1). These samples have been well characterized for major and trace element abundances and radiogenic isotopic compositions (full dataset and references in table S1), spanning nearly the full range of mantle radiogenic isotopic variability and including the most extreme isotopic compositions of different mantle end-members mentioned above (Fig. 2). This globally representative Mg isotopic dataset enables us to place tighter constraints on Mg isotopic compositions of different mantle end-members and to evaluate the recycling of surficial carbonates into the deep mantle.

**Fig. 1.**
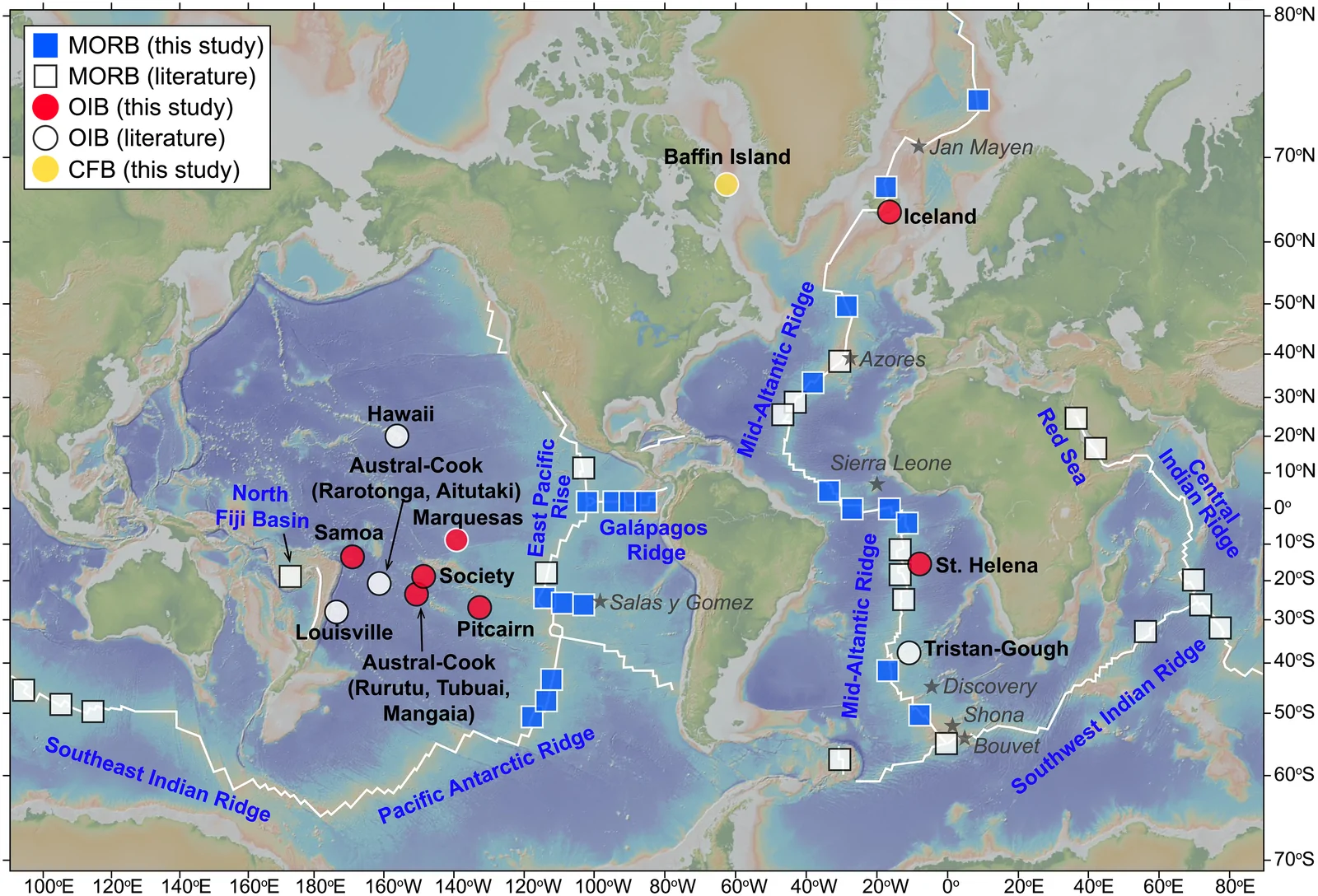
Map showing the distribution of sample localities of MORBs, OIBs, and the Baffin Island continental flood basalts (CFBs). Solid-colored symbols represent sample localities analyzed in this study, while black-outlined symbols denote localities with previously published Mg isotope data (*30*–*33*, *35*, *37*, *43*, *54*). Gray asterisks indicate the centers of hot spots adjacent to localities of the studied plume-influenced MORBs. Figure made with GeoMapApp (www.geomapapp.org) / CC BY 4.0 (https://creativecommons.org/licenses/by/4.0/).

**Fig. 2.**
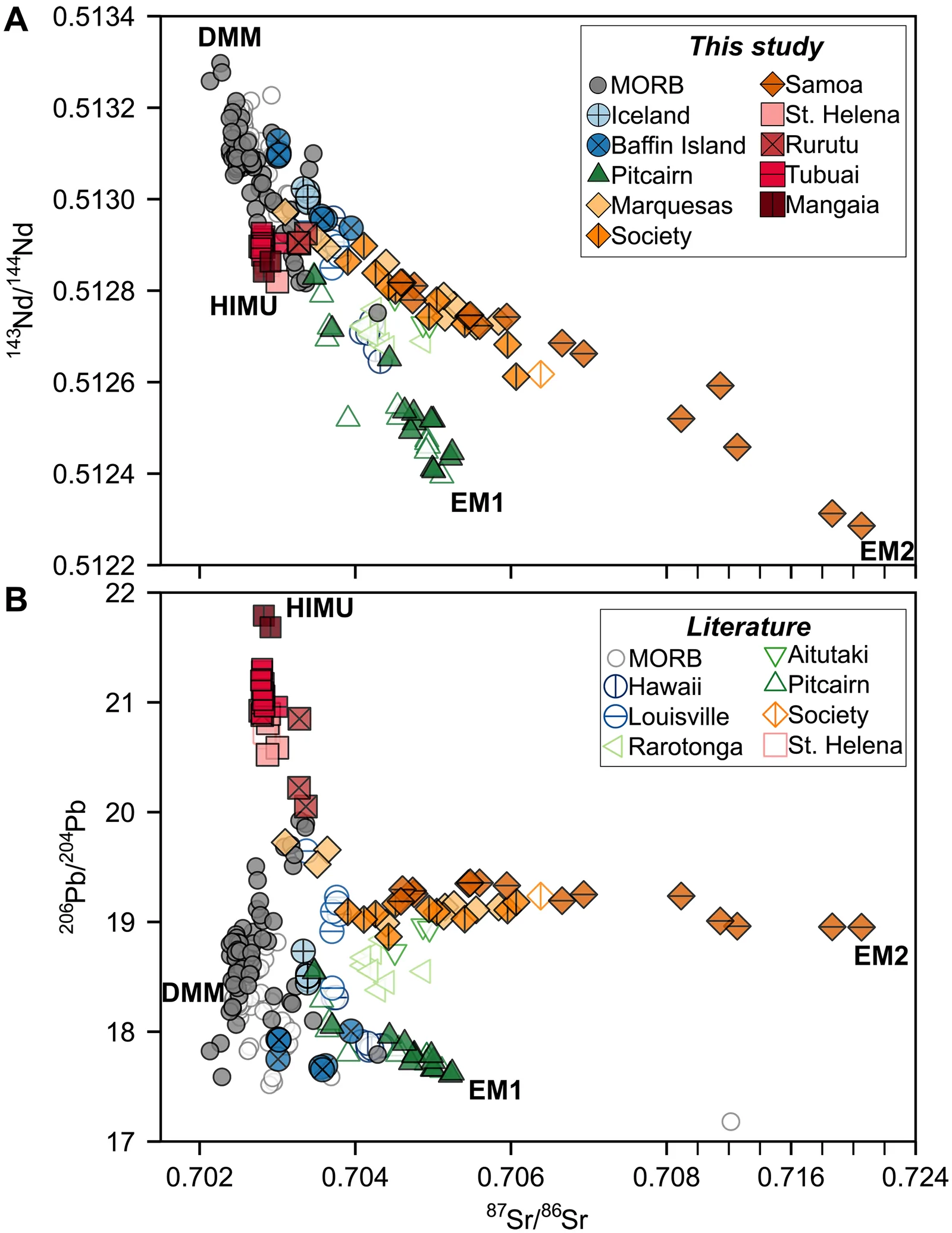
Radiogenic isotopic variability of the samples investigated for Mg isotopic compositions. Plots of ^143^Nd/^144^Nd (**A**) and ^206^Pb/^204^Pb (**B**) versus ^87^Sr/^86^Sr. Approximate positions of the DMM, HIMU, EM1, and EM2 mantle end-members (*48*, *85*) are plotted.

## RESULTS AND DISCUSSION

### Evaluation of postsource processes on Mg isotopes

The δ^26^Mg values range from −0.45 to −0.17‰ across both MORBs and OIBs. Notably, light Mg isotopic compositions are consistently observed in HIMU-, EM1-, and EM2-type OIBs, as well as a few plume-influenced MORBs (Fig. 3). Weathering or seafloor alteration typically shifts basalts toward high δ^26^Mg values, except when secondary carbonate precipitation occurs, which can instead enrich them in light Mg isotopes (*36*). Our samples display minimal evidence of carbonate alteration and exhibit no correlation between δ^26^Mg and proxies for alteration such as LOI, Ba/Rb, and Th/U (fig. S1). Therefore, low-temperature alteration is unlikely to account for their low δ^26^Mg values.

**Fig. 3.**
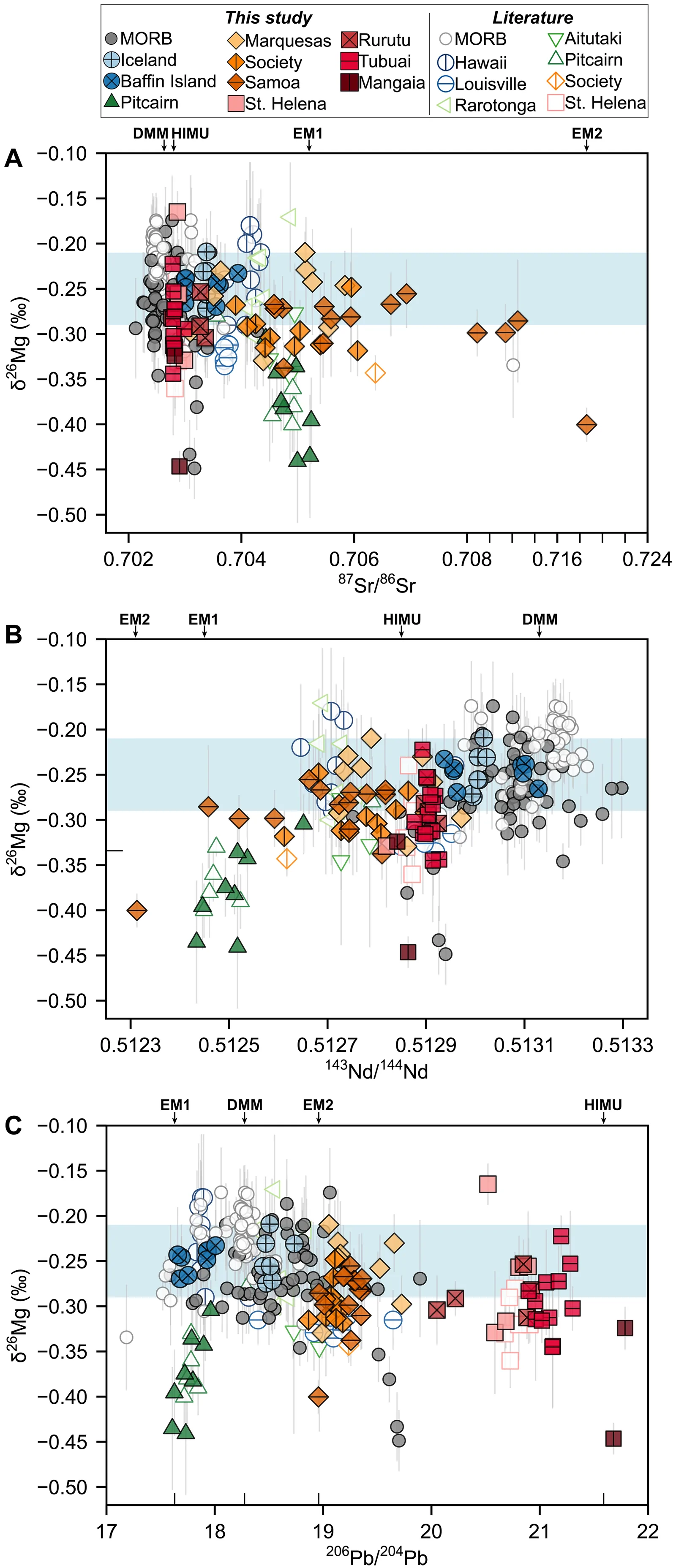
Relationships between δ^26^Mg value and radiogenic isotopic ratios in MORBs and hot spot lavas. Plots of δ^26^Mg versus ^87^Sr/^86^Sr (**A**), ^143^Nd/^144^Nd (**B**), and ^206^Pb/^204^Pb (**C**). The blue horizontal band denotes the δ^26^Mg range of BSE (*31*). Small arrows at the top of each panel indicate approximate Sr-Nd-Pb isotopic compositions of DMM, EM1, EM2, and HIMU mantle end-members (*48*, *85*). To minimize the effects of magmatic differentiation on Mg isotopes, only samples with MgO > 5 wt % are plotted in this and subsequent figures unless stated otherwise.

Magnesium isotope fractionation during high-temperature magmatic processes is limited (*14*). Nevertheless, subtle, yet systematic δ^26^Mg variations in oceanic basalts have been revealed in a few recent studies, which have been attributed to fractional crystallization (*34*, *37*). Olivine crystallization may increase melt δ^26^Mg by <0.1‰ (*37*, *38*) and cannot produce the observed low δ^26^Mg values. Segregation of isotopically heavy Fe-Ti oxides (e.g., titanomagnetite) at late-stage differentiation is a viable mechanism decreasing melt δ^26^Mg (*34*, *39*). However, most of isotopically light samples in our study have high MgO contents (>5.0 wt %) and exhibit an inverse correlation between MgO and TiO_2_. Moreover, δ^26^Mg values display no systematic relationship with MgO or TiO_2_ (Fig. 4 and fig. S2), indicating little influence of Fe-Ti oxide crystallization. Samples with MgO content of >20 wt % may represent mixtures of melt and accumulated olivine, but these samples are not significantly enriched in light Mg isotopes, ruling out olivine accumulation as a major cause of the δ^26^Mg variation. Consistent with these observations, modeling δ^26^Mg evolution during fractional crystallization and accumulation fails to reproduce the low δ^26^Mg values (Fig. 4).

**Fig. 4.**
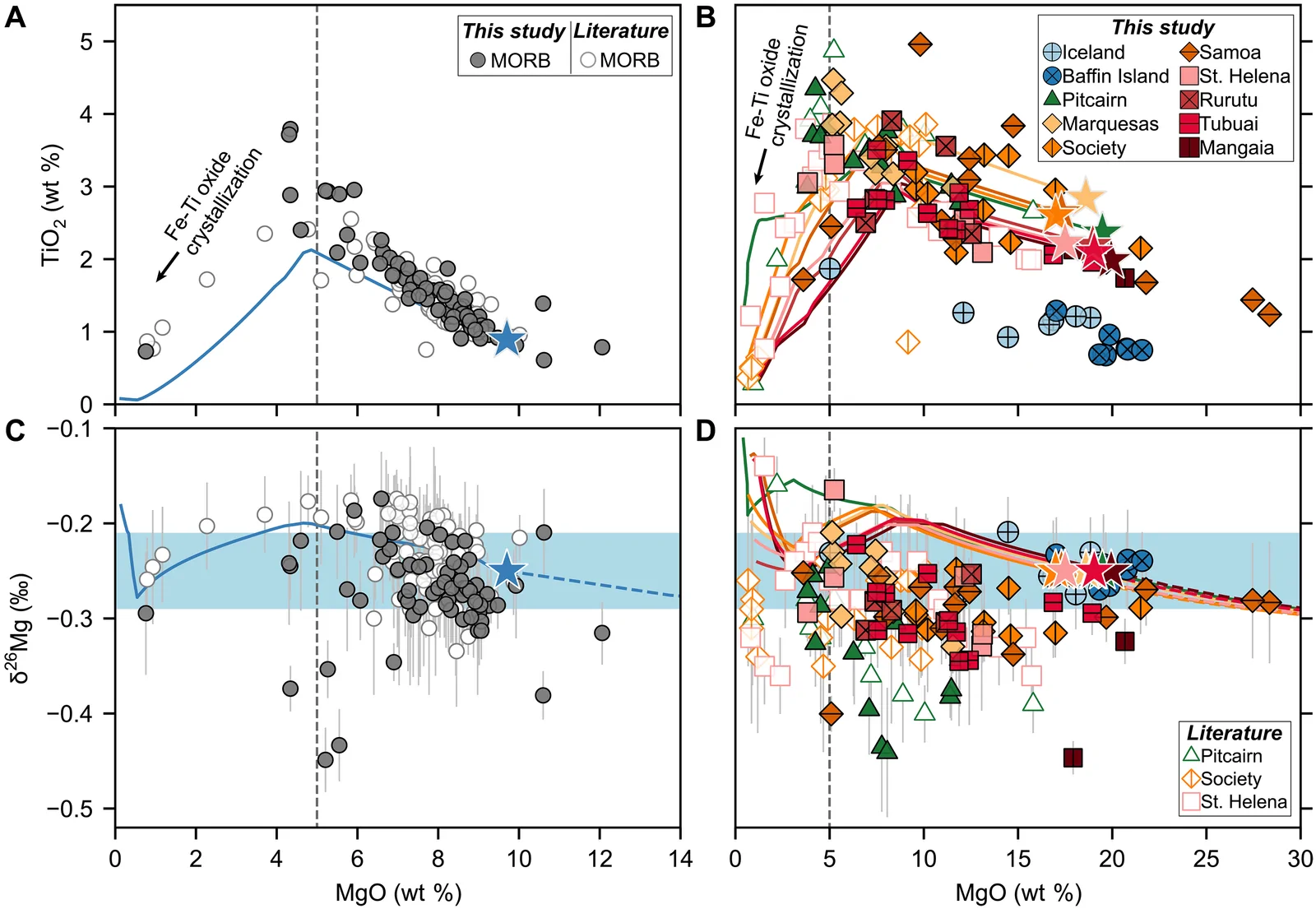
Modeling of Mg isotope fractionation in melts during fractional crystallization. (**A** and **B**) Plots of TiO_2_ versus MgO in MORBs (A) and hot spot lavas (B). (**C** and **D**) Plots of δ^26^Mg versus MgO in MORBs (C) and plume-related hot spot lavas (D). The vertical dashed line separates samples with Fe-Ti oxide crystallization from those with limited crystallization. The blue horizontal band denotes the δ^26^Mg range of BSE (*31*). Asterisks mark primary melts with assumed BSE-like δ^26^Mg values. Solid curves represent modeled melt δ^26^Mg evolution during fractional crystallization. Dashed curves represent mixing between primary melts with accumulated olivines with a fixed MgO content of 50 wt % and a δ^26^Mg value of −0.32‰. Modeling details are provided in table S6. All samples analyzed in this study are plotted.

Partial melting may also affect δ^26^Mg values of basaltic melts, particularly when garnet or spinel remains stable as a residual phase (*33*, *35*, *40*, *41*). Given that garnet prefers light Mg isotopes, melts derived from garnet-bearing mantle sources are expected to be isotopically heavier than their sources (*35*, *41*). If so, melts characterized by “residual garnet signatures,” such as elevated Dy/Yb ratios, should commonly exhibit high δ^26^Mg values (*42*). This is, however, at odds with the BSE-like to lower δ^26^Mg values in the OIB samples with high Dy/Yb ratios (Fig. 5). On the other hand, partial melting of a shallow, spinel-facies mantle may produce melts with slightly lower δ^26^Mg values than their sources, as spinel favors heavy Mg isotopes (*14*). This mechanism has been invoked to explain the lower δ^26^Mg values of some MORBs (*35*). Nevertheless, the lowest δ^26^Mg values in our MORB samples are significantly lower than those predicted by partial melting models, indicating that spinel-facies melting alone cannot account for the observed Mg isotopic variations.

**Fig. 5.**
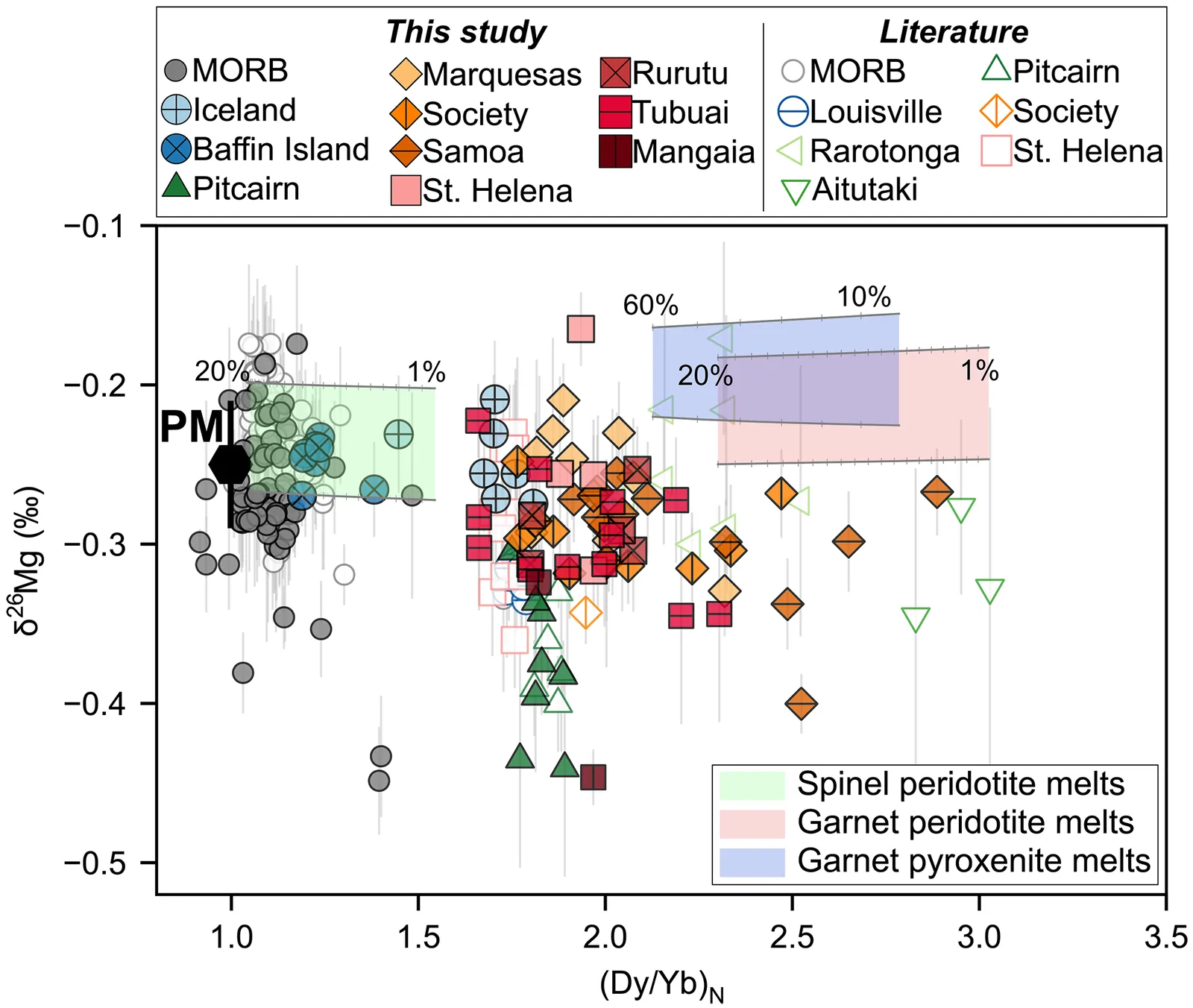
Modeling of melt Mg isotopic evolution during partial melting of different mantle lithologies. Plot of δ^26^Mg versus (Dy/Yb)_N_, where N refers to chondrite normalization (*86*). The green, purple, and red areas represent modeled melts of spinel peridotite, garnet peridotite, and garnet pyroxenite, respectively. The melting models assume an initial source δ^26^Mg of −0.25‰, with Δ^26^Mg_olivine − melt_ varying from 0.00 to −0.07‰. Modeling details are provided in tables S7 to S9. The hexagon denotes the mean δ^26^Mg of the primitive mantle (PM) (*31*), which has a chondritic Dy/Yb (*86*).

Another possibility recently proposed to explain δ^26^Mg variations in oceanic basalts is kinetic isotope fractionation related to Fe-Mg interdiffusion between melts and solid mantle during melt transport, whereby light Mg isotopes preferentially diffuse from peridotites into melts (*43*). However, well-documented diffusion profiles indicative of kinetic Mg isotope fractionation in natural samples are restricted to millimeter-to-centimeter mineral scales (*44*). Given the meter- to kilometer-scale volumes of basaltic melts, diffusion is unlikely to significantly alter their Mg isotopic compositions. Moreover, MORBs and OIBs lacking “recycled” signatures have δ^26^Mg values within the BSE range, indicating that any kinetic effects on melt Mg isotopic compositions are minor. We therefore conclude that the anomalously lower δ^26^Mg values extending beyond the BSE range are unlikely to result from postsource processes but instead primarily reflect isotopic heterogeneity in their mantle sources, as discussed below.

### Magnesium isotopic composition of the depleted lower mantle

Previous studies of non- or weakly metasomatized peridotites have yielded a relatively homogeneous δ^26^Mg value of −0.25 ± 0.04‰, regarded as representative of the bulk mantle (*31*, *41*), although lithospheric peridotites sample only the uppermost part of the mantle. By contrast, plume-related lavas can have elevated ^3^He/^4^He ratios compared with MORBs, widely interpreted as evidence for contributions from an ancient deep mantle reservoir that has remained relatively isolated from the upper mantle convection (*45*). High-^3^He/^4^He mantle plumes therefore provide valuable constraints on Mg isotopic composition of the lower mantle. The Iceland plume, sampled by lavas from Iceland and Baffin Island, is distinguished by the highest terrestrial ^3^He/^4^He ratios yet measured (*46*). Moreover, the Iceland and Baffin Island lavas analyzed here are characterized by low ^87^Sr/^86^Sr, high ^143^Nd/^144^Nd, and low ^206^Pb/^204^Pb ratios, making them among the most “incompatible element-depleted” plume lavas yet investigated for Mg isotopes (Fig. 2). These signatures indicate minimal involvement of recycled materials in their mantle sources (*47*). The homogenous and peridotitic mantle-like δ^26^Mg values of these samples therefore suggest that much of the lower mantle is isotopically indistinguishable from the upper mantle. This inference is further supported by the similarly BSE-like δ^26^Mg values observed in high-^3^He/^4^He (>30 Ra, where Ra denotes the atmospheric ^3^He/^4^He ratio) basalts from Samoa and Hawaii (fig. S3).

### Magnesium isotopic heterogeneity in OIB sources

OIB sampling mantle domains with recycled crustal signatures, in contrast to high-^3^He/^4^He mantle plumes, display greater Mg isotopic heterogeneity, driven primarily by samples with δ^26^Mg values below the BSE range. We therefore focus the following discussion on origins of these abnormally low δ^26^Mg values.

Partial melting of recycled oceanic crust in the form of pyroxenites at various pressures can partially explain δ^26^Mg variations in OIBs (*35*). However, as discussed above, most OIBs in our study were formed by melting under garnet stability conditions, which is expected to produce melts that are isotopically heavier than their sources. Consequently, partial melting of eclogitic or pyroxenitic sources with BSE-like δ^26^Mg values cannot generate isotopically light melts, unless these sources contain isotopically light recycled components.

The lowest δ^26^Mg values occur exclusively in OIBs with the most extreme HIMU, EM1, and EM2 compositions (Fig. 3). Moreover, samples with lower δ^26^Mg values tend to have higher D^Sr-Nd-Pb^ values (Fig. 6), a recycling index quantifying the contribution of recycled materials in mantle sources, defined as the distance between a hypothetical depleted mantle end-member and individual samples in a three-dimensional ^87^Sr/^86^Sr-^143^Nd/^144^Nd-^206^Pb/^204^Pb space; higher D^Sr-Nd-Pb^ values indicate a greater contribution of recycled crust (*48*). Together, these observations indicate that the recycled components responsible for radiogenic isotopic diversity of OIB mantle sources carry a common light Mg isotopic signature.

**Fig. 6.**
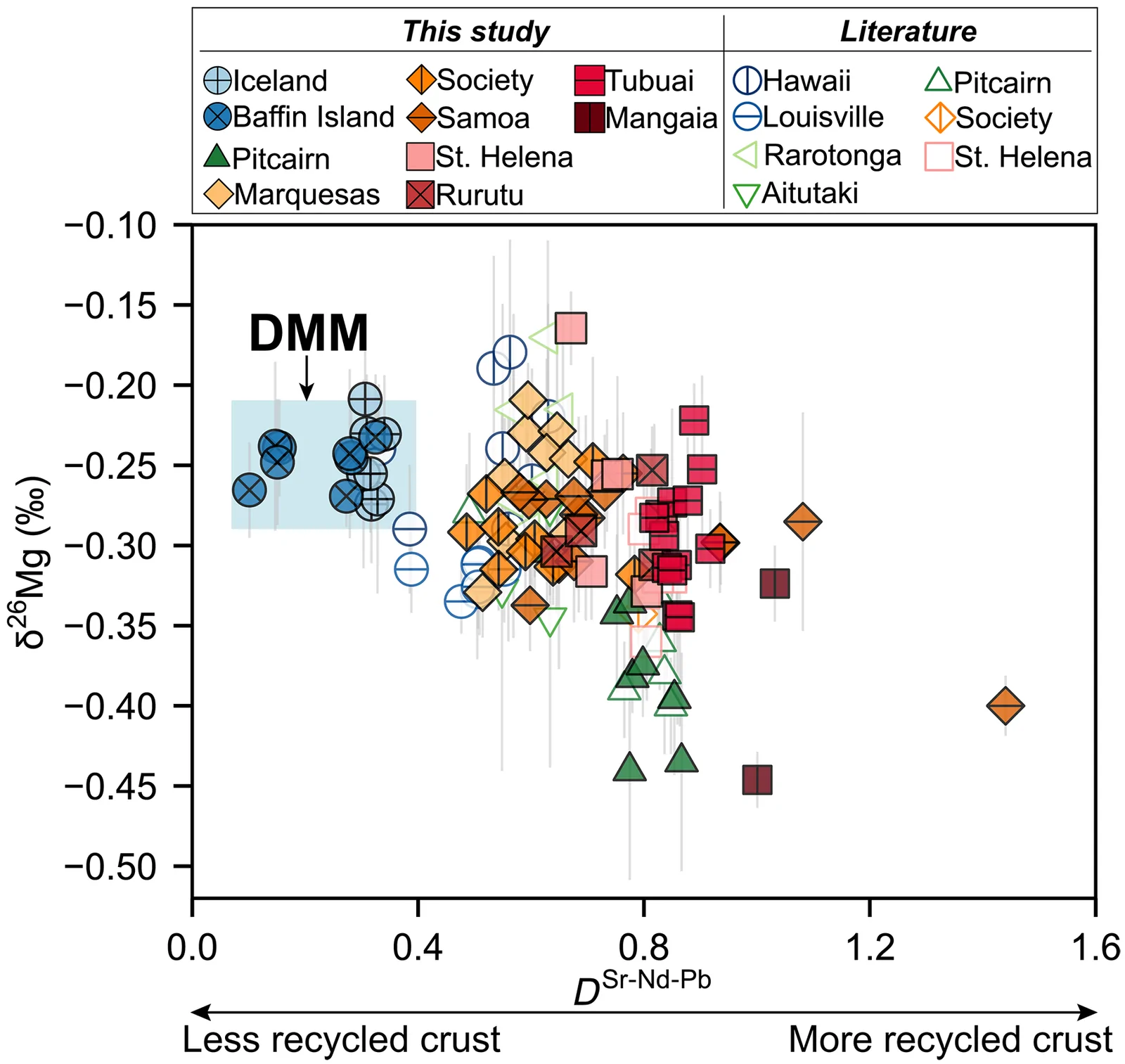
Coupled Mg and radiogenic isotope systematics of hot spot lavas. δ^26^Mg versus *D*^Sr-Nd-Pb^. *D*^Sr-Nd-Pb^ = ([(^87^Sr/^86^Sr)_sample_ – (^87^Sr/^86^Sr)_DM_]^2^/*X*^2^ + [(^143^Nd/^144^Nd)_sample_ – (^143^Nd/^144^Nd)_DM_]^2^/*Y*^2^ + [(^206^Pb/^204^Pb)_sample_ – (^206^Pb/^204^Pb)_DM_]^2^/*Z*^2^)^0.5^, where DM refers to a hypothetical isotopically depleted mantle end-member (^87^Sr/^86^Sr = 0.7025; ^143^Nd/^144^Nd = 0.5132; ^206^Pb/^204^Pb = 17.5) and *X*, *Y*, and *Z* represent differences in Sr, Nd, and Pb isotopic compositions, respectively, between the DM and the most extreme compositions observed in OIBs (^87^Sr/^86^Sr = 0.7205, ^143^Nd/^144^Nd = 0.5123, and ^206^Pb/^204^Pb = 22.0) (*48*); higher *D*^Sr-Nd-Pb^ values correspond to greater contribution of recycled crustal materials from EM1, EM2, and/or HIMU components. The compositional range of the DMM is shown as the blue area (*31*, *85*).

The isotopically light basalts are predominantly alkaline and formed by low degrees of mantle melting, as suggested by their high alkali index (*49*), whereas higher-degree melts such as tholeiitic lavas from the Icelandic and Hawaiian plumes generally have BSE-like δ^26^Mg values (Fig. 7). This contrast is consistent with preferential melting of fusible and isotopically light recycled components that contribute disproportionately to low-degree alkaline melts. Most subducted silicate rocks have BSE-like to higher δ^26^Mg values (*14*), making them unlikely to be the source of light Mg. Rodingites are a possible exception, with δ^26^Mg values as low as −1.5‰, but they are volumetrically scarce in subducting slabs and also commonly more enriched in heavy Ca isotopes (*50*), contrary to the MORB-like to lighter Ca isotopic compositions of OIBs (*10*, *51*). By contrast, sedimentary carbonates constitute the lightest terrestrial Mg reservoir (*14*) and represent the most plausible recycled component supplying light Mg to the OIB sources. This interpretation is also consistent with previous studies proposing that recycled carbonate-bearing crustal materials contribute to other isotopic features of OIBs, including light–Ca and heavy–Zn isotope enrichment (*10*, *12*).

**Fig. 7.**
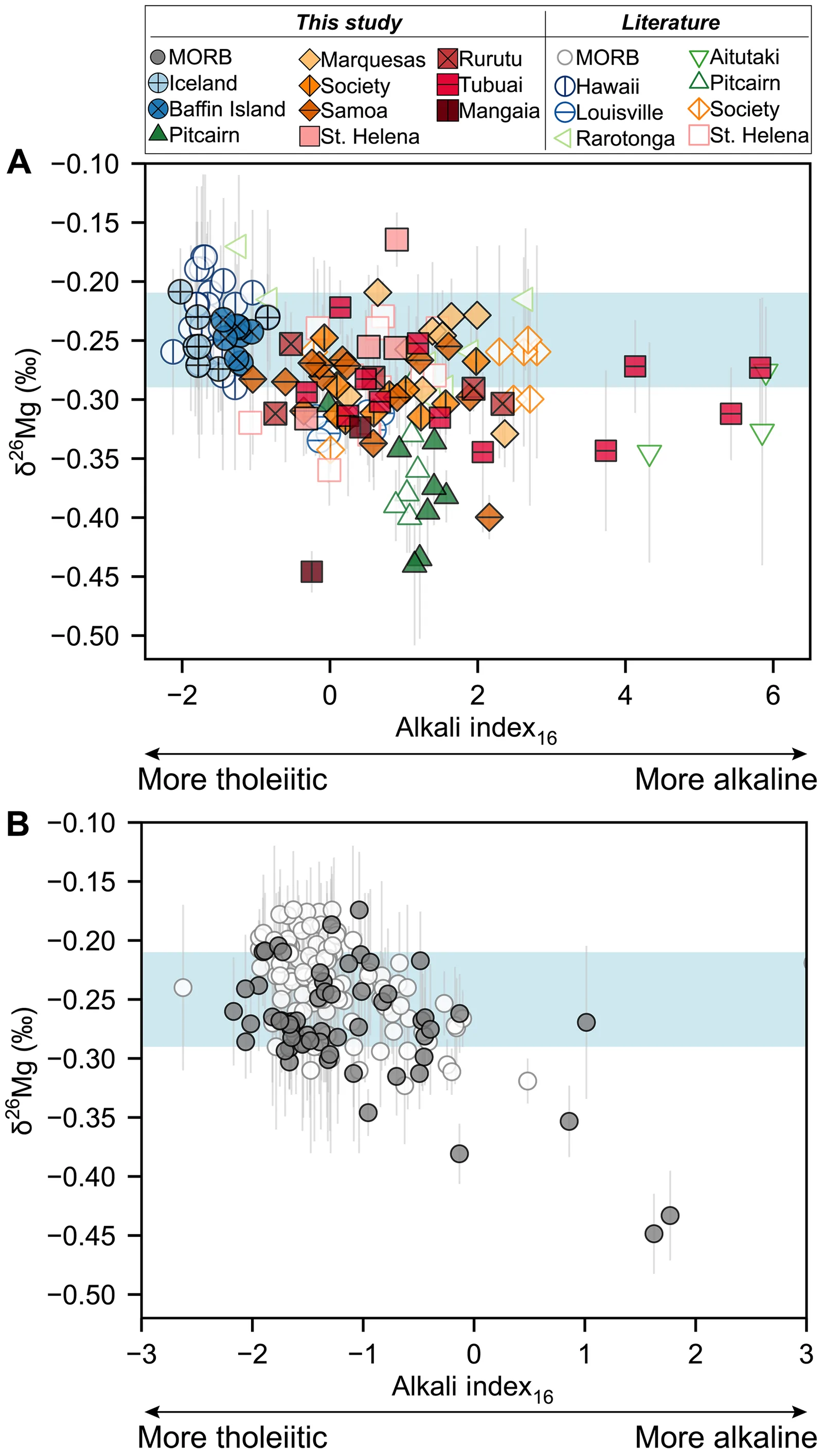
Relationship between δ^26^Mg value and alkali index. (**A**) Hot spot lavas. (**B**) MORBs. Alkali index_16_ is defined as (Na_2_O + K_2_O) − (0.37 × SiO_2_ −14.43), where the major oxides have been corrected for equilibrium olivine fractionation or addition back to a melt composition with 16 wt % MgO (*49*). A higher alkali index_16_ corresponds to lower degree of mantle melting and greater contribution of fusible components (e.g., carbonates and pyroxenites) to the melts relative to more refractory peridotites. The blue horizontal band denotes the δ^26^Mg range of BSE (*31*).

### Light Mg isotopic signatures in plume-influenced MORBs

Our Pacific-Antarctic Ridge samples, collected far from plume hot spots, exhibit homogeneous, BSE-like δ^26^Mg values (−0.25 ± 0.05‰), consistent with previous observations from depleted MORBs. These results further support a uniform Mg isotopic composition of the depleted upper mantle matrix. By contrast, MORB samples influenced by plume components display larger δ^26^Mg variations (−0.45 to −0.17‰) (Fig. 8), with lower δ^26^Mg values associated with higher alkali index (Fig. 7), similar to the pattern observed in alkaline OIBs.

**Fig. 8.**
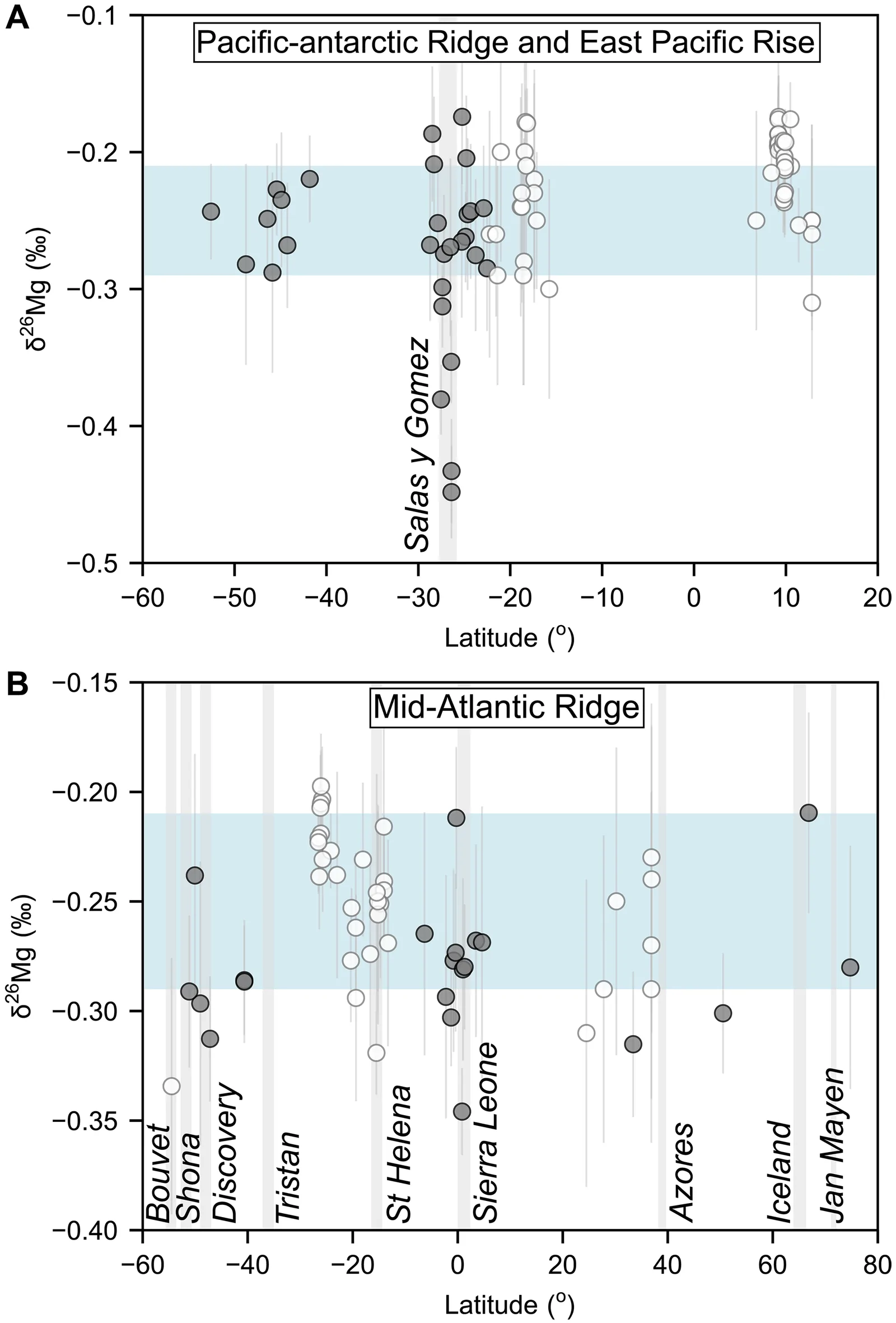
Latitude variations in δ^26^Mg values of MORBs. (**A**) The Pacific-Antarctic Ridge and East Pacific Rise. (**B**) The Mid-Atlantic Ridge. Vertical gray bands mark the latitudes of hot spots proximal to the ridges (*65*), and the horizontal blue band denotes the δ^26^Mg range of BSE (*31*).

The lowest δ^26^Mg values occur in basalts from the Easter-Salas y Gomez seamount chain section of the East Pacific Rise. These lavas have been interpreted as typical products of plume-ridge interaction, as manifested by systematic shifts in radiogenic isotopic compositions from DMM-like signature near the spreading centers to HIMU-EM1 mixed compositions with increasing distance from the ridge axis, reflecting progressively greater input from the Salas y Gomez plume (*52*, *53*). The lowest δ^26^Mg values are also observed in samples with stronger “incompatible element-enriched plume” signatures, including higher ^87^Sr/^86^Sr and ^206^Pb/^204^Pb, and lower ^143^Nd/^144^Nd ratios (fig. S4). This suggests that the plume component is the carrier of isotopically light Mg, plausibly derived from the recycled carbonate-bearing oceanic crust, as revealed in the OIBs. Similar observations have been recently reported in MORBs from the Mid-Atlantic Ridge (MAR) segment influenced by St. Helena plume, although their δ^26^Mg values extend only slightly below the BSE range, down to −0.32‰ (*54*). Our analyses also identify low δ^26^Mg values in MORB samples from the MAR segments influenced by the Azores, Sierra Leone, and Discovery plumes (Fig. 8). Together, these observations suggest that light Mg isotopic signatures are not restricted to individual plume systems but may be widespread among plume components sampled by both OIBs and plume-influenced MORBs.

### Magnesium isotopic constraints on origins of carbon in the lower mantle

Quantifying the mantle carbon budget remains challenging, because undegassed primary mantle-derived melts are rarely preserved. Current assessments therefore rely primarily on the ratios of CO_2_ relative to other gases (e.g., He, Ar, and N_2_) or to trace elements with comparable incompatibility to CO_2_ (e.g., Ba, Nb, and U). Despite the large heterogeneity in carbon contents among mantle sources feeding different volcanoes, results from these approaches generally indicate that the lower mantle sampled by OIBs (C = ∼300 to 600 ppm) contains substantially more carbon than the upper mantle represented by the DMM (C = ∼30 to 80 ppm) (*55*–*58*). Nevertheless, it remains unclear whether the “excess” carbon in OIB sources primarily derives from a primordial, less-degassed, and carbon-rich mantle domain formed early in Earth’s history or is instead attributable to surficial carbon recycled into the deep mantle over Earth’s long-term tectonic evolution.

Our findings of lower δ^26^Mg values in OIBs and plume-influenced MORBs relative to lavas derived from the depleted mantle suggest that recycled carbonates may contribute a non-negligible fraction of the carbon budget in the lower mantle. To quantify the contribution of recycled carbonates to the δ^26^Mg variations in these lavas, we use a Monte Carlo mixing model in which melts of carbonate-bearing oceanic crust mix with peridotite-derived melts, taking into account the possible ranges of their MgO contents and δ^26^Mg values (modeling details are provided in Materials and Methods). It is important to note that this approach estimates the amount of carbonate-derived Mg incorporated into the melts, rather than the proportion of carbonates in the mantle source. The subducted carbonates entering the lower mantle source are assumed to have dolomitic compositions, as dolomites are more resistant to dissolution during slab dehydration than calcites (*22*). If the carbonate end-member is assigned a δ^26^Mg value equal to the mean of dolomites (−1.87 ± 0.44‰) sampled over the full range of geologic ages, then the model requires $2.0_{-0.7}^{+0.7}$ and $8.5_{-1.9}^{+3.2}$ wt % carbonate (equivalent to $0.95_{-0.3}^{+0.3}$ and $4.1_{-0.9}^{+1.5}$ wt % CO_2_ in melts) to reproduce the average (−0.30‰) and lowest (−0.45‰) δ^26^Mg values of alkaline OIBs, respectively (Fig. 9 and fig. S5). The occasional presence of mass-independent sulfur isotope anomalies in OIBs suggests that their mantle sources may contain recycled materials of Archean-Paleoproterozoic ages (*59*). As dolomites have progressively lower δ^26^Mg values from the Paleoproterozoic to the present because of the secular evolution of the seawater composition (*60*), we additionally evaluated a Paleoproterozoic dolomite end-member with a δ^26^Mg value of −1.11 ± 0.22‰. Under this assumption, higher carbonate proportions of $3.6_{-1.0}^{+1.5}$ and $17.1_{-3.9}^{+6.9}$ wt % (corresponding to $1.7_{-0.5}^{+0.7}$ and $8.2_{-1.9}^{+3.3}$ wt % CO_2_ in melts) are required to reproduce the same average and lowest δ^26^Mg values (Fig. 9 and fig. S6). The inferred melt CO_2_ contents exceed those measured in OIB melt inclusions (<2 wt %) (*61*), but they are close to the parental CO_2_ contents of alkaline OIBs (∼3 to 11 wt %) constrained from experimental melt CO_2_-SiO_2_ systematics (*56*).

**Fig. 9.**
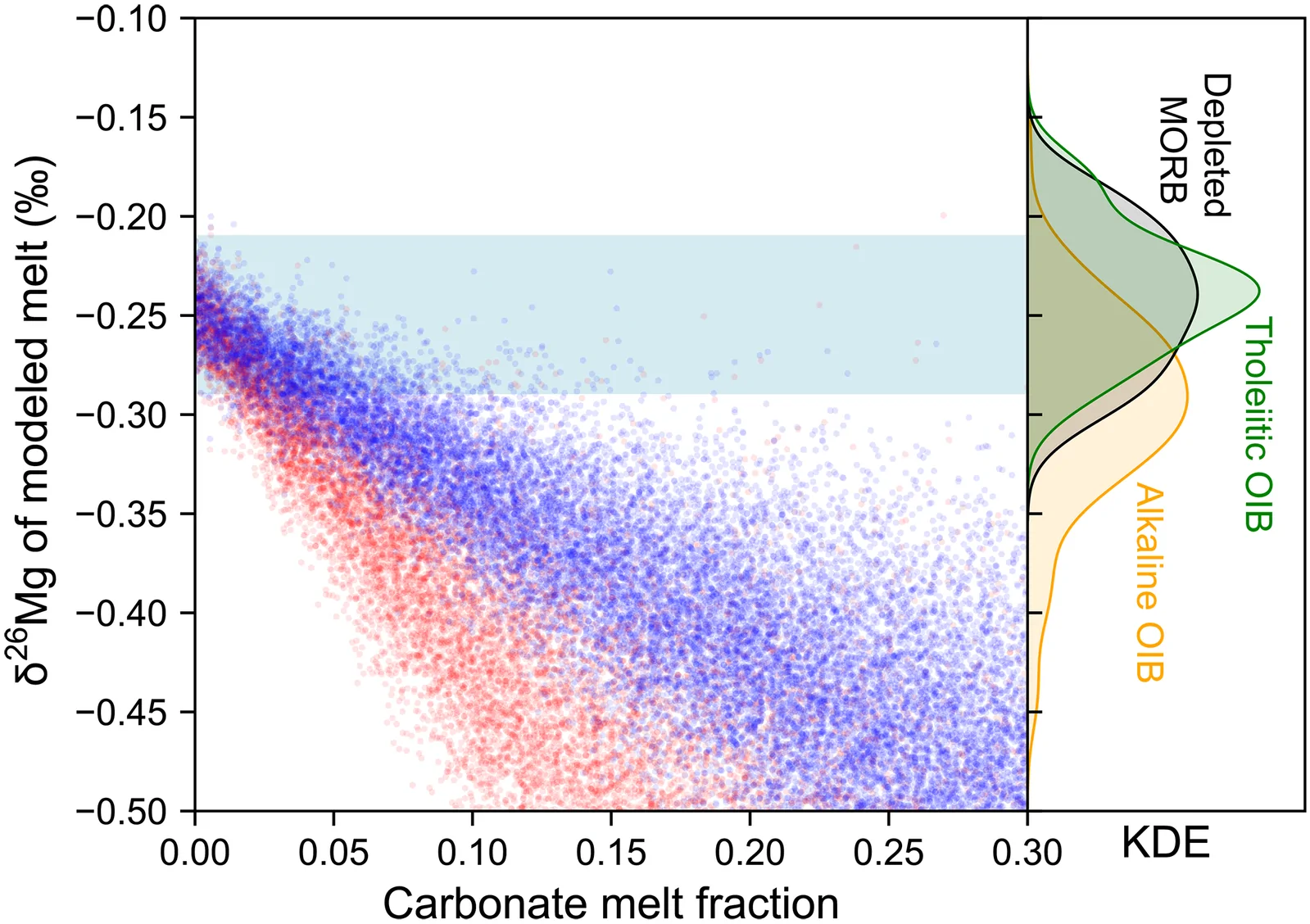
Comparison of modeled carbonated mantle melts with MORBs and OIBs. Red dots show Monte Carlo simulations of mixing between silicate melts derived from peridotite-eclogite sources and carbonate melts, where the carbonates are assumed to have a δ^26^Mg value equal to the mean composition of carbonates sampled over the full range of geologic ages. Blue dots show equivalent simulations in which the carbonate end-member is restricted to Paleoproterozoic carbonates. Kernel density estimates (KDEs) of δ^26^Mg for depleted MORBs, tholeiitic OIBs, and alkaline OIBs are shown on the right for comparison. The complete dataset is provided in table S5. The blue horizontal band denotes the δ^26^Mg range of BSE estimate (*31*).

Mantle CO_2_ contents can be approximated as the melt CO_2_ contents multiplied by the melt fraction as CO_2_ is highly incompatible during mantle melting. The degree of melting for alkaline OIBs inferred from highly incompatible element abundances and comparisons between the major element compositions of experimental carbonated peridotite melts and natural lavas is likely ∼1 to 5% (*56*, *62*), although this estimate depends on the assumed composition of the mantle. Applying this melting range, together with the melt CO_2_ contents estimated from the average δ^26^Mg values of alkaline OIBs, yields CO_2_ contents of ∼95 to 477 ppm (i.e., ∼26 to 130 ppm C) and ∼170 to 850 ppm (i.e., ∼46 to 232 ppm C) in their mantle sources for the two carbonate end-member models, respectively.

A key assumption of our model is that carbon remains coupled with Mg during subduction and transport into the lower mantle. Any decarbonation during slab subduction could lead to an overestimation of carbon fluxes into the mantle when using Mg or other metal isotopes. For example, under cold geotherms and reducing mantle conditions, carbonates may react with the surrounding mantle to form diamonds (*63*). If these newly formed diamonds detach from the slab, Mg may continue to be transported into the deep mantle while carrying a “ghost carbonate” light isotopic signature (*30*). This process could explain the decoupling between mantle-like δ^13^C values of ferropericlase-bearing diamonds and the extremely low δ^26^Mg values of their ferropericlase inclusions (*25*).

Despite these uncertainties, Mg isotopes provide a first-order constraint on the upper limit of the recycled carbon budget in the mantle. The growing Mg isotopic evidence for recycling of sedimentary carbonate-bearing materials into the lower mantle indicates that the deep mantle is an important reservoir for recycled carbon, underscoring the need to include deep mantle reservoirs in estimates of carbon fluxes through the deep carbon cycle.

### Geodynamic implications of mantle Mg isotopic heterogeneity

The mantle contains ∼99.9% of Earth’s Mg inventory; its Mg isotopic composition should strongly buffer perturbations introduced by recycling of crustal materials. Detectable deviations from the mantle δ^26^Mg value therefore require large Mg isotope fractionations during surface processes, such as carbonate precipitation (*14*), efficient transport of these signatures into the deep mantle, and long-term preservation despite billions of years of mantle convection. The close association between low δ^26^Mg values and recycling-sensitive radiogenic isotope signatures in OIBs indicates that mantle convection is considerably less efficient than would be required to homogenize the Mg isotopic composition of Earth’s largest Mg reservoir. Recycled crustal lithologies can persist as incompletely mixed carbonated eclogite and pyroxenite domains, consistent with the “marble-cake” mantle model (*64*). Their lower solidus temperatures allow them to contribute disproportionately to mantle-derived magmas although they represent only a minor fraction of the source (*62*), highlighting lithology-selective melting as a key mechanism by which mantle plumes sample and expose long-lived mantle heterogeneity.

## MATERIALS AND METHODS

### Samples

Most of the MORB glass samples were obtained from the Schilling Volcanic Glass collection at the University of Rhode Island, including 21 samples from the Mid-Atlantic Ridge, 11 samples from the Galápagos Ridge, and 23 samples from the East Pacific Rise. These samples were previously collected from ridges that are proximal to mantle plumes to evaluate the influence of plume activities on the geochemistry of MORBs (*65*, *66*). They display larger variations in radiogenic isotopes than typical depleted MORBs, indicating the involvement of enriched plume components (*52*, *53*, *66*). An additional 10 MORB samples are from the Pacific-Antarctic Ridge, which were collected during the PACANTARCTIC-2 cruise. Previous studies suggested that their geochemical and isotopic variations are unrelated to plume activities but more likely reflect a marble-cake mantle assemblage composed of a residual mantle component and a recycled oceanic crust component (*67*, *68*).

The OIB and continental flood basalt samples are from seven hot spot regions: Austral-Cook, St. Helena, Pitcairn, Samoa, Marquesas, Society, and Iceland. Among them, 19 samples from the Austral-Cook islands of Rurutu, Mangaia, and Tubuai (*69*–*72*) and 6 samples from the St. Helena island (*73*–*75*) represent typical HIMU-type lavas with ^206^Pb/^204^Pb of >20; 13 samples from the Pitcairn islands (*76*, *77*) display the strongest EM1 signatures and are characterized by ^206^Pb/^204^Pb as low as 17.611; 9 samples from the Marquesas islands (*78*), 15 samples from the Samoa islands (*79*–*83*), and 10 samples from the Society islands (*77*) have substantial variability in ^87^Sr/^86^Sr and ^143^Nd/^144^Nd, extending toward extreme EM2 compositions with ^87^Sr/^86^Sr reaching up to 0.7205; 7 samples from northwest Iceland (Vestfirðir) (*48*) and 8 picrites from Baffin Island (*47*) belong to products of the Iceland hot spot track that has depleted mantle-like Sr-Nd-Pb isotopic ratios and the highest terrestrial mantle-derived ^3^He/^4^He ever measured (*46*). Though not technically OIBs because of eruption as flood basalts in a continental setting, the Baffin Island samples investigated here have been rigorously examined to avoid continental contamination and are considered as “the least crustally contaminated” lavas that have depleted light rare earth element patterns, mantle-like Ce/Pb and Nb/Th ratios, and mantle-like oxygen isotopic ratios (*47*).

### Magnesium isotopic analysis

Sample digestion, column chromatography, and instrumental analysis were carried out at the Isotope Laboratory of the University of Washington, Seattle, following the established protocols (*84*). A brief description of this method is present below. Around 1 to 3 mg of whole-rock powders containing 50 to 100 μg of Mg were successively digested in Teflon beakers with a mixture HF and HNO_3_ (3: 1, v/v), aqua regia, and concentrated HNO_3_. After complete dissolution, sample solutions were dried down and refluxed with 1 N HNO_3_ in preparation for column chromatography. To separate Mg from other matrix elements, an aliquot of solution containing ∼20 μg of Mg was passed through a column filled with 2 ml of Bio-Rad AG50W-X8 (200 to 400 mesh) cation exchange resin twice with 1 N HNO_3_ as the eluent. The total yield of Mg is better than 99%, and the whole procedural blank contains <10 ng of Mg, which can be considered negligible compared to the loaded Mg mass.

Magnesium isotopic ratios were measured on a Nu Plasma II multicollector inductively coupled plasma mass spectrometer using the standard-sample bracketing method to correct the instrumental mass bias. Both the purified sample solutions and bracketing Mg standards were diluted to a concentration of 400 parts per billion (ppb) in 3% HNO_3_. Solutions were introduced into the plasma using a Cinnabar spray chamber and a MicroMist micro-uptake glass concentric nebulizer with an uptake rate of 0.1 ml/min. All isotopes were analyzed in a low-resolution mode, which yielded a ^24^Mg signal intensity of 2 to 3 V/100 ppb. The final data are reported in δ^26^Mg and δ^25^Mg notation against the DSM3 Mg standard: δ26, 25Mg (‰)=[Mg26, 25/Mg24sample(Mg26, 25/Mg24)DSM3−1]×1000. The average of repeat analyses is reported as the Mg isotopic composition of a given sample. The analytical uncertainty is presented as 2SD of multiple analyses on bracketing standards during an analytical session, which is better constrained and more conservative than 2SD on the basis of a few repeat analyses on individual sample solutions.

Accuracy and reproducibility were assessed using several reference materials that were processed through sample dissolution and column chemistry alongside the studied samples. These reference materials include two peridotite standards (DTS-1 and PCC-1), four basalt standards (BCR-2, BIR-1a, BHVO-1, and BHVO-2), one andesite standard (AGV-1) and two in-house reference materials (San Carlos olivine and Hawaiian seawater). Their δ^26^Mg values are reported in table S2 and agree well with the recommended values within uncertainty (*14*). Repeated analyses of both samples and standards throughout this study yielded δ^26^Mg values reproducible within ±0.04‰, better than the long-term external uncertainty of the laboratory [±0.07‰; (*84*)].

### Quantifying recycled carbonate contributions to Mg isotopic variations in melts

The δ^26^Mg values of peridotite and the silicate portion of oceanic crust are broadly BSE-like (*14*). The only component capable of significantly lowering the δ^26^Mg value of the resulting melt is the carbonate itself. Thus, the model can be simplified as mixing between carbonate-derived Mg and silicate-derived Mg from melts of basaltic oceanic crust and peridotites (silicate-rich sediments are not involved because of their low Mg contents). The proportion of carbonate melts (*f*_carb._) can be constrained using the isotope mass-balance equation

$$\begin{array}{c} \delta^{26}{\text{Mg}}_{\text{mixed melt}}=\\ \frac{\left(f_{\text{carb}.}\times \delta^{26}{\text{Mg}}_{\text{carb}.}\times {\text{MgO}}_{\text{carb}.}+f_{\text{OC}}\times \delta^{26}{\text{Mg}}_{\text{OC}}\times {\text{MgO}}_{\text{OC}}+f_{\text{peri}.}\times \delta^{26}{\text{Mg}}_{\text{peri}.}\times {\text{MgO}}_{\text{peri}.}\right)}{\left(f_{\text{carb}.}\times {\text{MgO}}_{\text{carb}.}+f_{\text{OC}}\times {\text{MgO}}_{\text{OC}}+f_{\text{peri}.}\times {\text{MgO}}_{\text{peri}.}\right)} \end{array}$$

where OC refers to melts of oceanic crust and peri. refers to melts of peridotites. To fully explore the possible combinations of end-member compositions and mixing proportions, we performed a Monte Carlo simulation consisting of 50,000 random realizations. In each iteration, δ^26^Mg values and MgO contents for the carbonate, oceanic crust, and peridotite melt end-members were randomly sampled from their respective probability distributions, which are summarized in table S10, and the resulting δ^26^Mg value of the mixed melt was calculated using the mass-balance equation above. To estimate the most likely *f*_carb._ for each mixed-melt δ^26^Mg value, we used the median *f*_carb._ of all Monte Carlo realizations that produced the corresponding target δ^26^Mg value within ±0.005‰; uncertainties are reported as the 25th and 75th percentiles.
