# Supplementary material for: Surficial carbonates in mantle plume sources revealed by magnesium isotopes
Source: Sci Adv. 2026 Oct 9;12(41):eaee9247. doi: 10.1126/sciadv.aee9247 (PMC13651868; doi:10.1126/sciadv.aee9247)
Supplement: Supplementary file 1 [file sciadv.aee9247_sm.pdf]

Science Advances
AAAS

Supplementary Materials for

Surficial carbonates in mantle plume sources revealed by magnesium isotopes

Ze-Zhou Wang *et al.*

Corresponding author: Ze-Zhou Wang, wzz@cugb.edu.cn

Sci. Adv. **12**, eaee9247 (2026)
DOI: 10.1126/sciadv.aee9247

The PDF file includes:

Figs. S1 to S6
Legends for tables S1 to S10
Legends for movies S1 and S2

Other Supplementary Material for this manuscript includes the following:

Tables S1 to S10
Movies S1 and S2

Supplementary figures

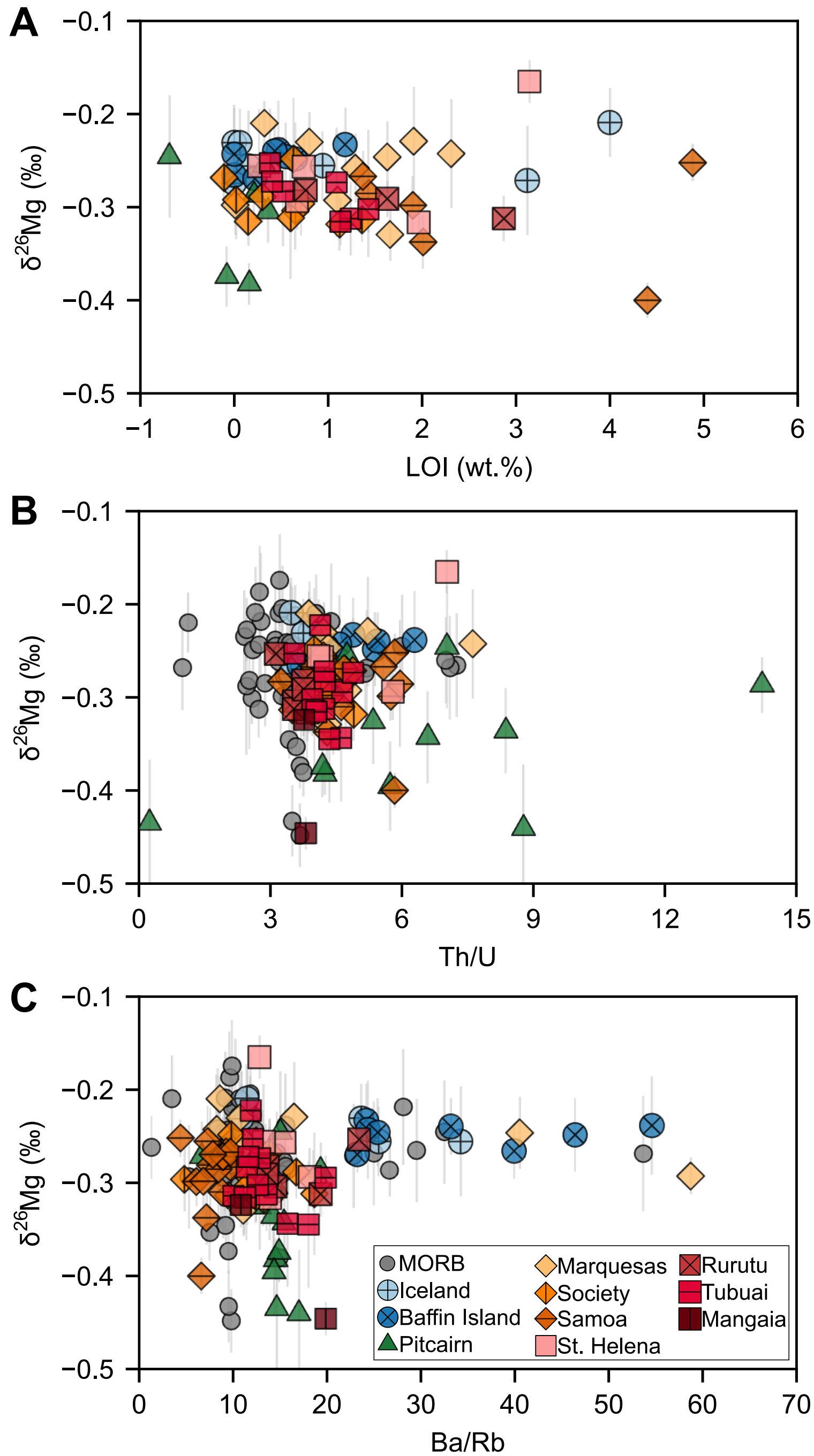

Figure S1: Relationships between δ^{26}Mg value and proxies for low-temperature alteration. Plots of δ^{26}Mg versus loss on ignition (LOI) (**A**), Th/U (**B**), and Ba/Rb (**C**). Not all samples have LOI data. Low-temperature alteration typically increases Th/U and Ba/Rb owing to the higher mobility of U^{6+} relative to Th and of Rb relative to Ba in fluids. Samples with Th/U > 4.5 or Ba/Rb > 30 are considered potentially affected by alteration (*1*).

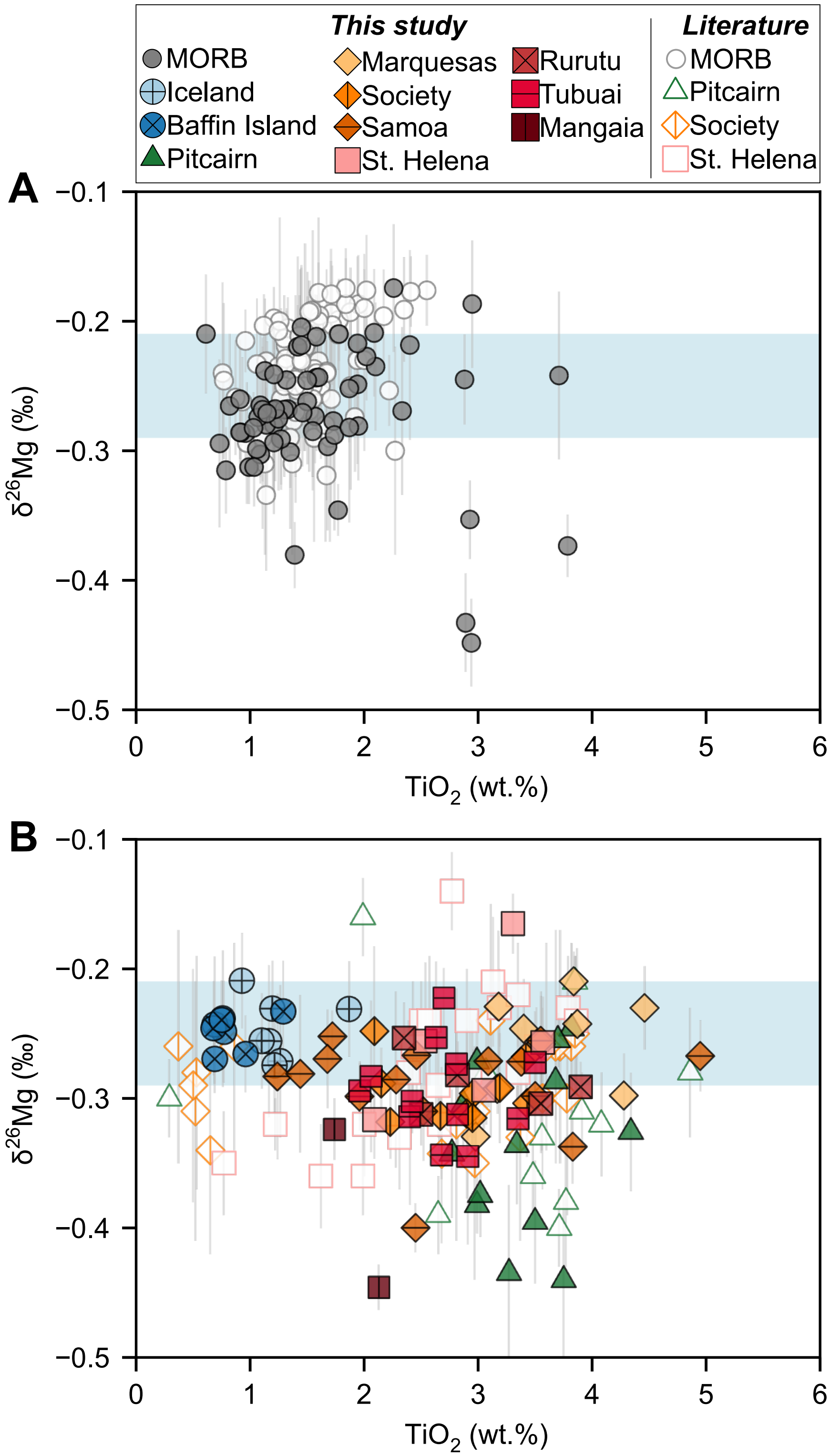

Figure S2: Relationship between δ^{26}Mg value and TiO_2. (**A**) Hotspot lavas. (**B**) MORBs. The lack of obvious correlation suggests limited influence of Fe-Ti oxide crystallization on Mg isotopic variation. The blue horizontal band denotes the δ^{26}Mg range of BSE (*2*).

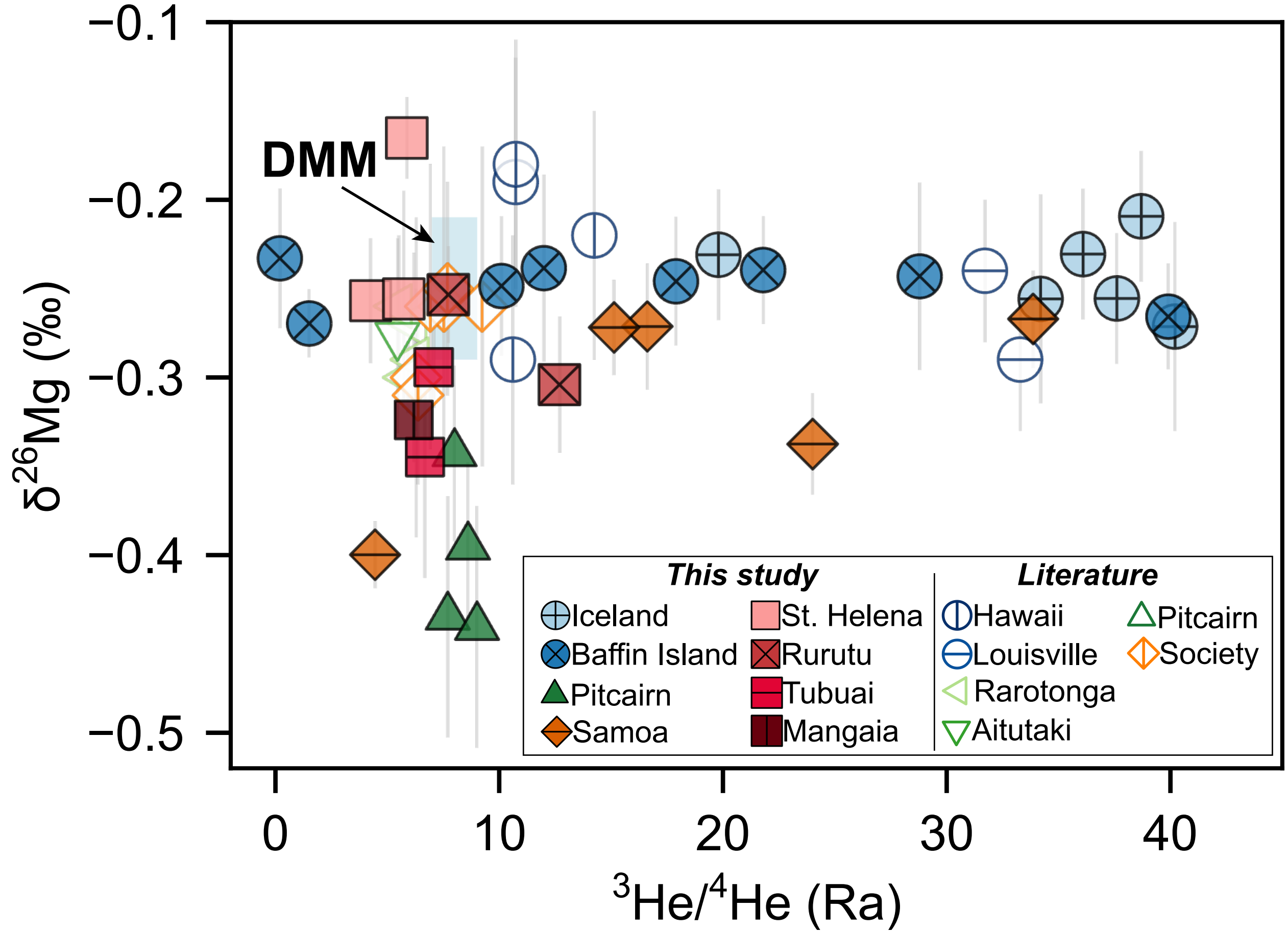

Figure S3. Relationship between δ^{26}Mg value and ^{3}He/^{4}He ratio. Ra refers to the atmospheric ^{3}He/^{4}He ratio. Compositions of the DMM are plotted for comparison (*2, 3*). The limited δ^{26}Mg variation in plume lavas with ^{3}He/^{4}He > 30 Ra indicates a relatively homogeneous Mg isotopic composition of the lower mantle matrix.

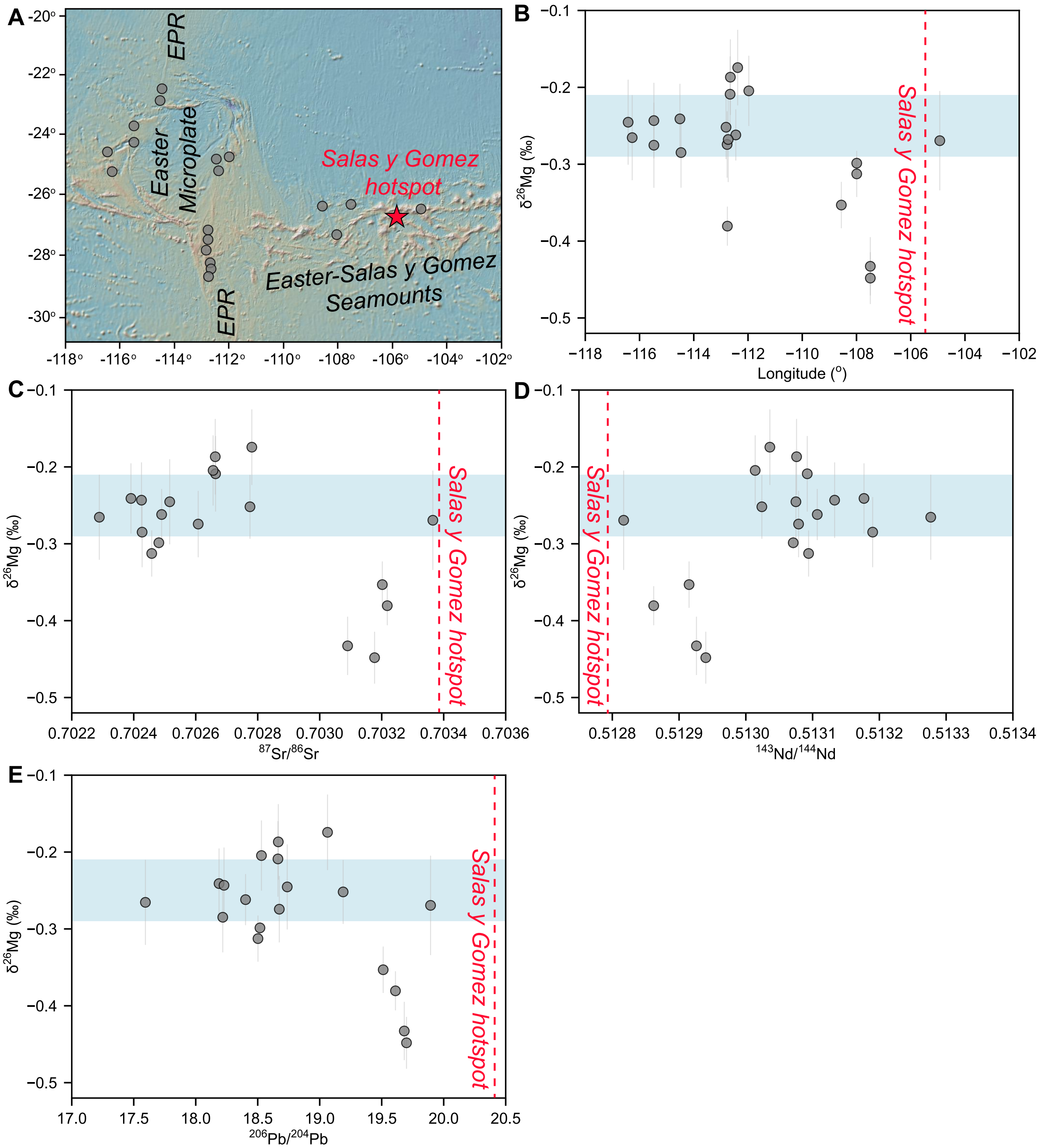

Figure S4: Relationships between Mg and radiogenic isotopes in lavas from the Easter-Salas y Gomez seamount chain section of the East Pacific Rise (EPR). (**A**) Map showing sampling localities. Figure made with GeoMapApp (www.geomapapp.org) / CC BY 4.0 (https://creativecommons.org/licenses/by/4.0/). (**B–E**) Variations in δ^{26}Mg value versus longitude (**B**), ^{87}Sr/^{86}Sr (**C**), ^{143}Nd/^{144}Nd (**D**), and ^{206}Pb/^{204}Pb (**E**). Red dashed lines refer to the approximate location and Sr-Nd-Pb isotopic compositions of the Salas y Gómez hotspot (*4, 5*). The isotopically lighter samples are

located closer to the Salas y Gómez hotspot and have more enriched Sr-Nd-Pb isotopic compositions. The blue horizontal band denotes the δ^{26}Mg range of BSE (*2*).

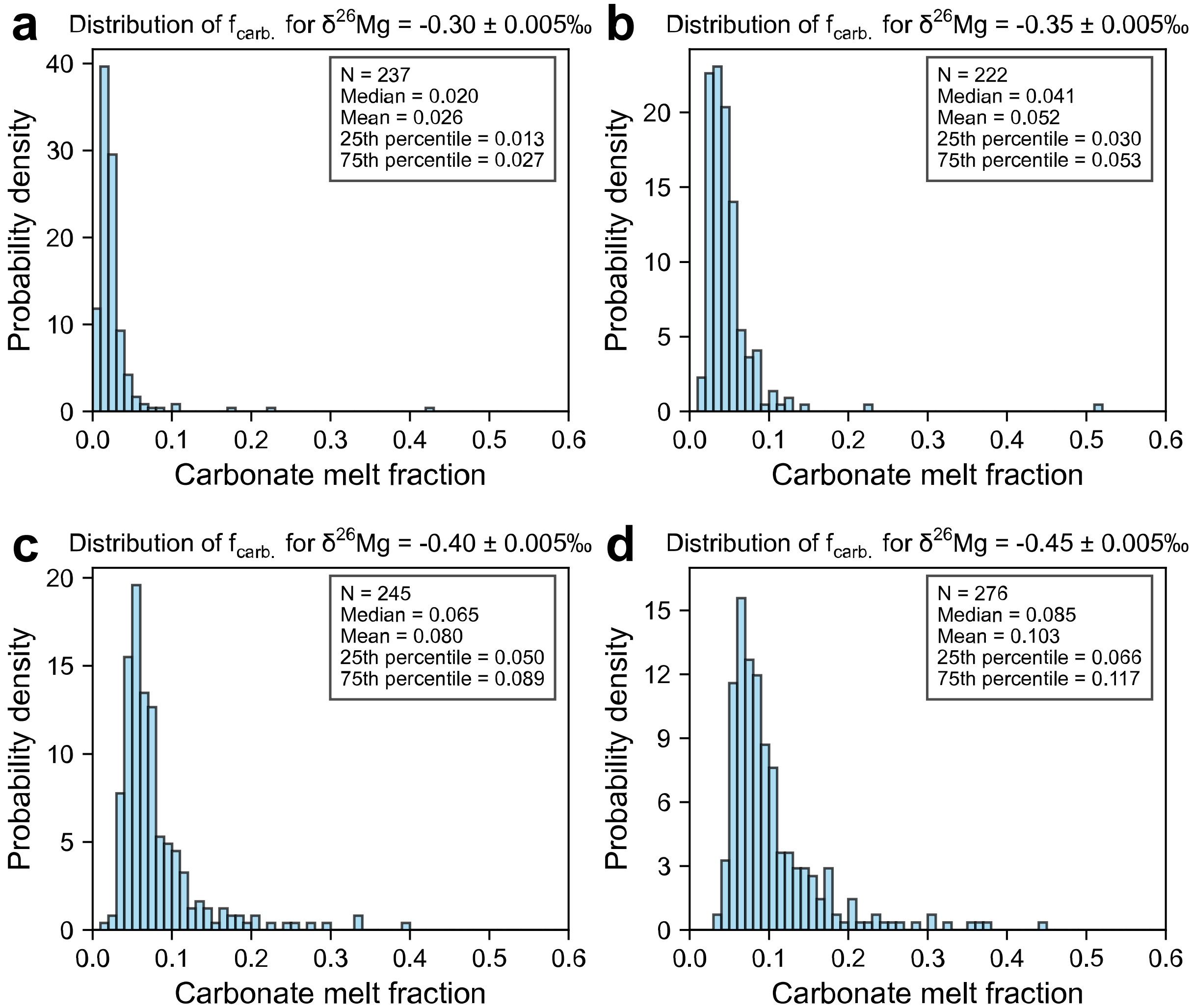

Figure S5: Probability distributions of carbonate melt fraction ($f_{carb.}$) required to reproduce different δ^{26}Mg values of modeled melts. The models assume that the carbonate endmember has a δ^{26}Mg value equal to the mean δ^{26}Mg of dolomites sampled over the full range of geologic ages. Panels (**A–D**) display the distributions of $f_{carb.}$ required to match melts with δ^{26}Mg values of (**A**) -0.30 ± 0.005‰, (**B**) -0.35 ± 0.005‰, (**C**) -0.40 ± 0.005‰, and (**D**) -0.45 ± 0.005‰. Each panel gives the number of accepted Monte-Carlo solutions (N), as well as the median, mean, 25th percentile, and 75th percentile of the calculated f_{carb}.

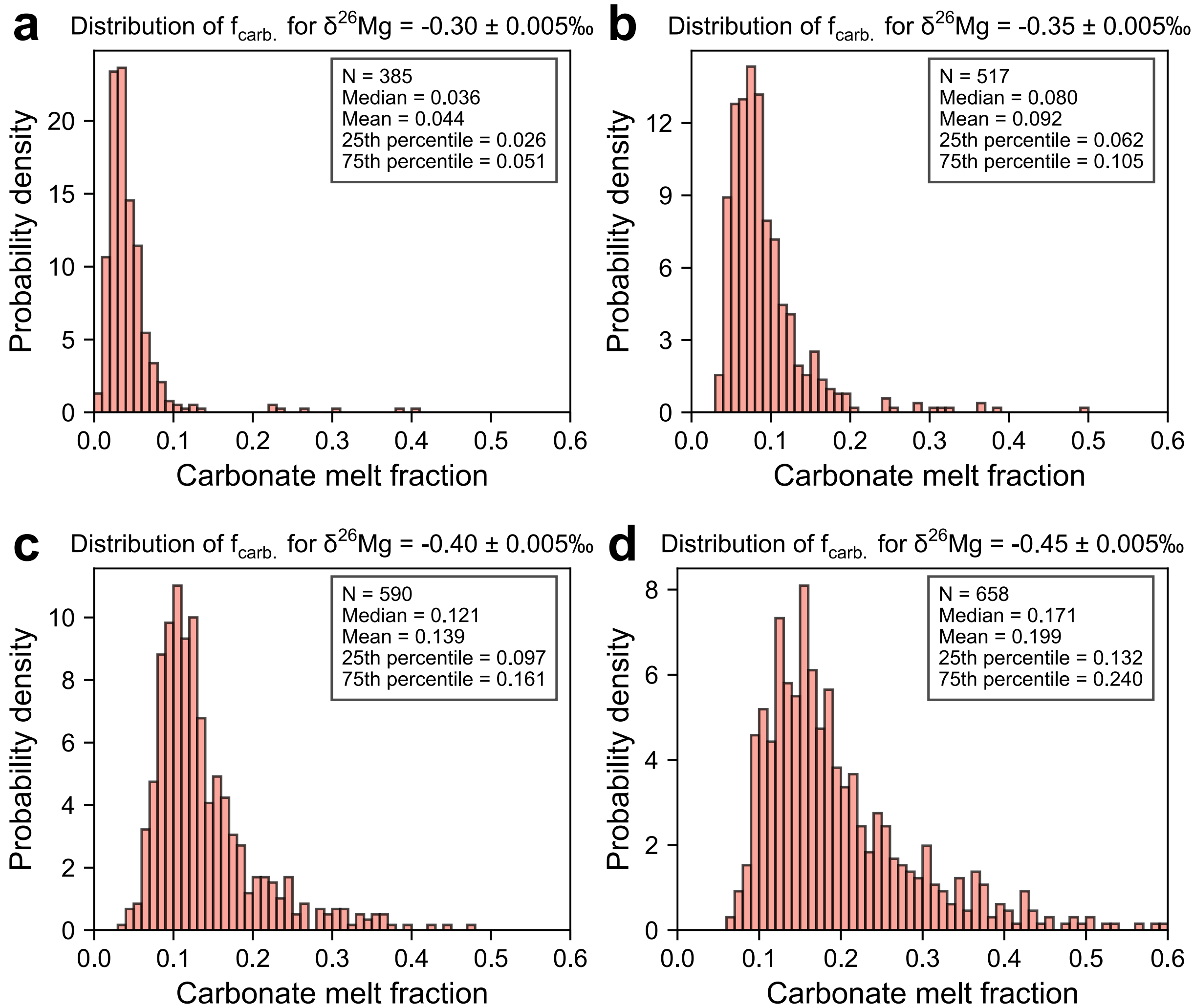

Figure S6: Probability distributions of carbonate melt fraction ($f_{carb.}$) required to reproduce different δ^{26}Mg values of modeled melts. The models assume that the carbonate endmember has a δ^{26}Mg value equal to the mean δ^{26}Mg of dolomites of Paleoproterozoic ages. Panels (**A–D**) display the distributions of $f_{carb.}$ required to match melts with δ^{26}Mg values of (**A**) -0.30 ± 0.005‰, (**B**) -0.35 ± 0.005‰, (C) -0.40 ± 0.005‰, and (**D**) -0.45 ± 0.005‰. Each panel gives the number of accepted Monte-Carlo solutions (N), as well as the median, mean, 25th percentile, and 75th percentile of the calculated f_{carb}.

Supplementary Excel tables

Table S1: Major and trace elemental and Sr-Nd-Pb isotopic compositions of samples investigated in this study.

Table S2: The Mg isotopic compositions of geological reference materials analyzed in this study.

Table S3: The Mg isotopic compositions of MORB samples investigated in this study.

Table S4: The Mg isotopic compositions of hotspot lava samples investigated in this study.

Table S5: Compiled Mg isotopic dataset for mid-ocean ridge basalts (MORBs) and ocean island basalts (OIBs).

Table S6: Modelling results of evolution of Mg isotopic compositions during fractional crystallization of MORB and OIB melts.

Table S7: Modelling results of evolution of melt δ^{26}Mg and Dy/Yb during partial melting of a spinel peridotite source.

Table S8: Modelling results of evolution of melt δ^{26}Mg and Dy/Yb during partial melting of a garnet peridotite source.

Table S9: Modelling results of evolution of melt δ^{26}Mg and Dy/Yb during partial melting of a MORB-like garnet pyroxenite source.

Table S10: Compositions of melt endmembers used in the Mg isotope mass-balance equation.

Supplementary movies

Movie S1: Three-dimensional distribution of MORBs and hotspot lavas in a Mg-Sr-Nd isotopic space.

Movie S2: Three-dimensional distribution of MORBs and hotspot lavas in a Mg-Sr-Pb isotopic space.
